# Study on methane degradation by microbial agents based on chelating wetting agent carriers

**DOI:** 10.1038/s41598-024-66399-x

**Published:** 2024-07-04

**Authors:** Yumiao Han, Lianman Xu, Runjie Zhang, Jin Lv, Fengshuo Yang, Chen Ma

**Affiliations:** School of Environment, Liaoning University, Shenyang, 110036 China

**Keywords:** Methane-oxidizing bacteria, Chelating wetting agent, Carriers, Methane degradation, Gas pressure, Coal mine, Biotechnology, Environmental sciences, Natural hazards, Engineering

## Abstract

Due to the low permeability characteristics of the deep gas-containing coal seam, the conventional prevention and control measures that cannot solve the problems of gas outbursts are unsatisfactory for the prevention and control of the coal and gas outbursts disaster. Therefore, in this study, a strain of methane-oxidizing bacteria M_07_ with high-pressure resistance, strong resistance, and high methane degradation rate was selected from coal mines. The growth and degradation abilities of M_07_ in chelating wetting agent solutions to assess its adaptability and find the optimal agent-to-M07 ratio. It provides a new method for integrating the reduction of impact tendency and gas pressure in deep coal mines. The experimental results show that M_07_ is a Gram-positive bacterium of the genus *Bacillus*, which has strong resistance and adaptability to high-pressure water injection. By degrading 70 mol of methane, M_07_ produces 1 mol of carbon dioxide, which can reduce gas pressure and reduce the risk of gas outbursts in coal mines. As the experiment proves, the best effect was achieved when the M_07_ concentration of the chelating wetting agent was 0.05%. The methane-oxidizing bacteria based on the chelating wetting agent as carriers prove a new prevention and control method for the integrated prevention and control of coal and gas outbursts in coal mines and also provide a new idea for microbial application in coal mine disaster control.

## Introduction

Traditional methane control methods, such as water injection technology combined with a mine ventilation system^1^. Emerging methane control methods, such as the thermal stimulation of coal seam enhanced gas production technology^2,3^, methane release dynamics reliability analysis^4,5^, and methane factor risk indicator system assessment methods^6^. The above techniques can reduce methane content and control methane emissions to a certain extent. However, there are drawbacks, such as high power consumption and high cost during use. In contrast, injecting methane-oxidizing bacteria (MOB) into coal seams or applying them to coal walls based on their ability to degrade methane and convert it to carbon dioxide and water is a more effective method to prevent and control coal and gas outbursts. In the case of coal and gas outbursts disaster coal seams^7^, relying solely on gas extraction^8^ may not be sufficient to reduce the gas content in the coal seams. Therefore, choosing to add MOB to the water and inject it into the coal seam to reduce the possibility of coal and gas outbursts^9^. However, in the context of deep low-permeability coal seams, gas extraction is challenging, and there exists a higher risk of coal and gas outbursts. This is due to the low porosity of low-permeability coal seams restricting the entry of MOB into the micropores of the coal body^10^, which leads to the limited effectiveness of MOB in controlling coal and gas outbursts. To enhance the efficacy of MOB in preventing coal and gas outbursts disasters, it becomes necessary to improve the permeability of the coal seams, increase the injection of MOB into the coal seams, and reduce the gas pressure and content within the coal seams.

Currently, MOB is used to oxidize and decompose methane in mine gas, effectively controlling the methane concentration and reducing the risk of coal and gas outbursts^11,12^. Guo et al.^13^ demonstrated the use of MOB to decompose or convert gas into harmless substances, leading to a reduction in gas pressure and content within the coal seam. From a safety perspective, Zhao et al.^14^ analyzed the potential hazards of MOB and their metabolites, offering valuable insights for assessing the safety of microbial water injection agents. Under low methane concentration conditions, Zhang et al.^15^ investigated the degradation rate of MOB, showing a consistent decrease in methane concentration with the action of these bacteria. The relationship between methane reduction and degradation time followed a certain exponential function, indicating the potential for developing and applying this technology to prevent excessive methane concentration in outflow streams through the air roadway. Moreover, Liu et al.^16^ explored the applicability of surfactants in enhancing the degradation of coal body methane by MOB. Their results indicated that the addition of suitable surfactants could improve the efficiency of methane degradation by these bacteria, providing valuable information for optimizing carrier materials and microbial applications. These studies collectively highlight the effectiveness of injecting MOB into coal seams to reduce gas pressure and content, and effectively reduce the risk of coal and gas outbursts. However, it is important to note that conventional MOB and the parameters of their injection into the coal rock formation massif may not be effective in preventing and controlling compound dynamic disasters in deep coal seams.

At present, the majority of MOB used in coal seams water injection employ nitrate mineral salts medium (NMS medium) as a carrier, while some researchers utilize the absorbent sponge^17^ as a carrier. However, no research has identified carrier materials suitable for MOB to treat gas in deep low-permeability coal seams. Hence, there is a need to explore novel carrier materials for MOB.

To improve the solution permeability in low-permeability coal seams, wetting agents are usually using various methods of hydraulic fracturing or wetting agents added in fluid (to reduce the surface tension). Chen et al.^18^ present the evaluation of the effect of SDBS surfactant on coals, results show that SDBS surfactant greatly increased the coal wetting performance. Wang et al.^19^ investigated the influence of monomeric surfactants and compound surfactants at various concentrations on coal’s wettability, The results showed that when the surfactant solution concentration was greater than the critical micelle concentration, the coal’s wettability was significantly enhanced as the surfactant concentration increased. Gou et al.^20^ studied the impact of surfactants on the wettability of various coals and noted significant wettability variations among coal samples. Li et al.^21^ examined the effects of different surfactants on coal wettability and observed significant differences in their impact on experimental coal samples. Although surfactants as wetting agents can improve the water injection effect of coal seams by increasing the surface wetting agent of coal seams, there are deficiencies in the water injection effect for deep low-permeability coal seams due to the weak effect on porosity. As a result, the research team developed a chelating wetting agent^22,23^, Xu et al.^24,25^ investigate the mechanisms of tetrasodium iminodisuccinate (IDS) and SDBS separately, discovering that chelating agent can react directly with easily precipitated ions, forming a stable cyclic compound, preventing easily precipitated ions react with other substances. Therefore, the process of water injection to join a certain amount of chelating agent can prevent easily precipitated ions react with the surfactant. The chelating agent interacts with minerals like calcite and dolomite, leading to mineral dissolution, increased coal pore size and volume, decreased specific surface area, and improved pore connectivity, resulting in enhanced water injection. It is apparent that incorporating substances such as chelating agents and surfactants can improve coal seams' permeability. Nevertheless, water injection agents can expel coal seam gas through water injection pressure, which may pose a problem with methane leakage into the roadway. The chelating wetting agent formed by the composite of surfactant and chelating agent is used as a carrier, which can enhance the wettability of the deep low-permeability coal seam and improve the porosity by utilizing its properties. On the one hand, it can strengthen the effect of water injection and exhaust^26^. On the other hand, it can realize the large-area contact between MOB and methane in coal seams and promote its capture and degradation of coal seams gas^27^, so that to realize the effective control of gas concentration.

To tackle the challenges of preventing and controlling compound dynamic disasters in deep low-permeability coal seams, this study screened a strain of MOB with robust resistance and high pressure from coal samples. The strain was then blended with the chelating wetting agent to assess its compatibility with the agent, and relevant experiments were conducted to determine the optimal ratio between the two. The resulting microbial agent utilizing the chelating wetting agent as its carrier effectively reduces gas pressure and coal body impact tendency in the coal seam. Thus, this study proposes a novel approach for preventing and controlling compound dynamic disasters, including rock bursts and coal and gas outbursts in deep coal seams.

## Materials and methods

### Experimental materials

To more accurately screen MOB with a high degradation rate, high-pressure resistance, and suitability for coal seam water injection, the samples collected this time were chosen as coal samples from a coal mine in Henan, China^28^. The experiment employed NMS medium^29^ and basal medium^30^ for the screening of desired MOB.

*NMS medium* MgSO_4_·7H_2_O, 1.000 g/L; KNO_3_,1.000 g/L; Na_2_HPO_4_·12H_2_O, 0.717 g/L; KH_2_PO_4_, 0.272 g/L; CaCl_2_·6H_2_O, 0.200 g/L; NH_4_Cl, 0.250 g/L; 0.1% (v/v) of trace element solution.

*Liquid basal medium* Beef paste, 0.9 g; Peptone, 3.0 g; NaCl, 1.5 g; Distilled water, 300 mL; (Solid basal medium requires the addition of 6 g agar).

*Methane* purchased from Shenyang Guangtai Gas Co., Ltd, purity 99.999%.

**Figure 1:**
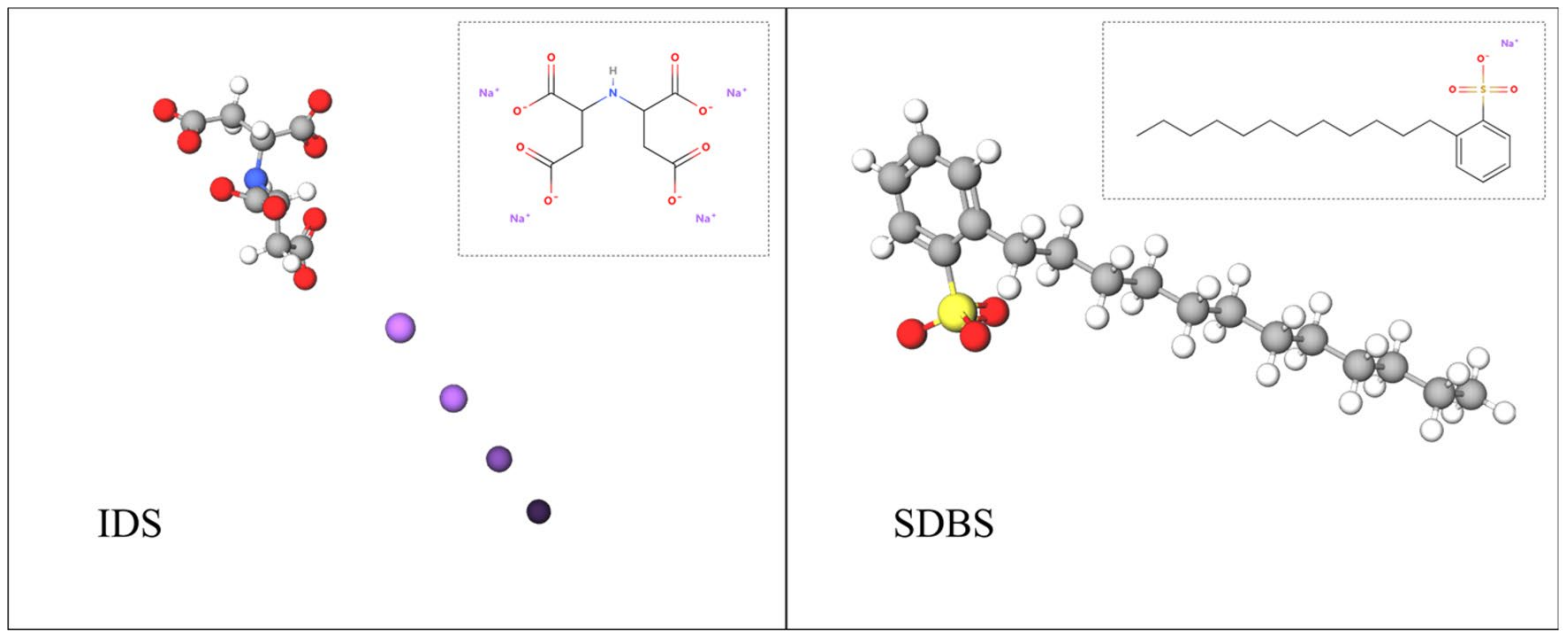
Molecular formulae of IDS and SDBS.

According to the research of Lianman Xu, Kaixuan Lu, Yajing Li, and others in this group^31,32^ on coal seam water injection additives to reduce the impact tendency of deep low-permeability coal seams, two monomers, namely, IDS, a chelating agent, and SDBS (Fig. 1), a surfactant, were mixed in a volume ratio of 1:1 to act as chelating wetting agents in the present experiment.

### Strain screening and identification

#### Samples collection

The coal mine samples collected in the field were immediately placed in sealed bags and refrigerated at – 20 °C. They were then promptly transported back to the laboratory for further processing. Each sample, weighing 2 g, was added to 100 mL of sterilized distilled water. After shaking for 30 min and allowing it to settle for 10 min, the upper suspension was carefully extracted and labeled for each sample.

#### Enrichment culture

Extract a 5 mL water sample from each treated sample and introduce it into a saline bottle containing 50 mL NMS medium. Ensure that proper labels are attached to each sample. The bottles were filled with 20% (v/v) methane and placed in a constant temperature oscillating incubator at 36 °C and 150 r/min for culture. The gas inside the bottles was changed daily to ensure adequate methane and oxygen levels. After 7 days, the medium in the bottle would become turbid. The enrichment solution was transferred to an equal amount of fresh NMS medium, following a 10%(v/v) inoculum volume, and then continued with the above operation. This process needs to be repeated three times for transfer.

#### Isolation and purification

**Figure 2:**
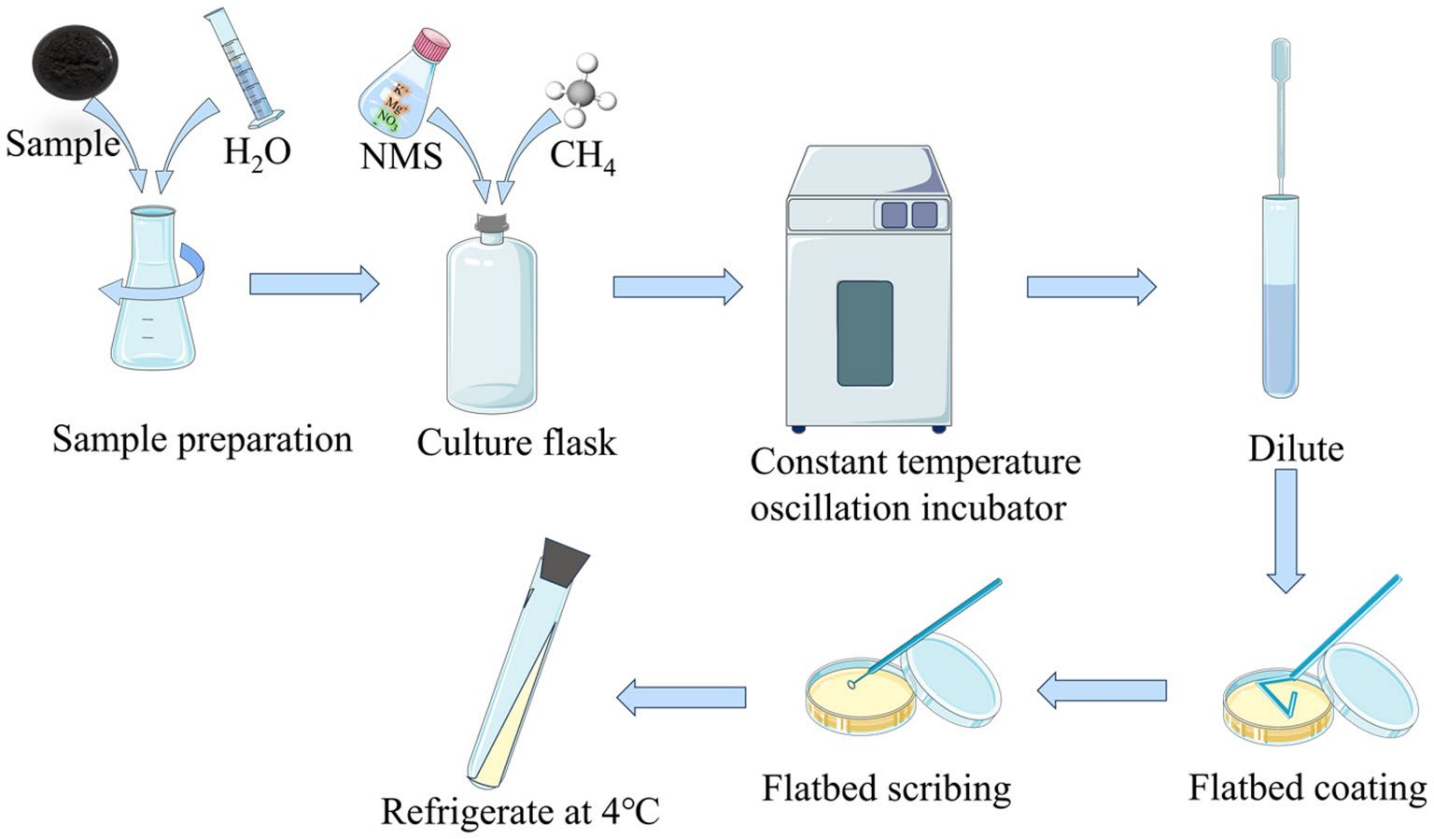
Schematic diagram of strain enrichment, isolation and purification process.

We dilute the enriched solution tenfold, then take the appropriate dilution of the bacterial solution, apply it to the solid basal medium by plate spreading method, put it into a constant temperature incubator, and keep it at 36 °C for cultivation. After the colonies grow out, we can perform simple staining to observe the morphology of the colonies and use the inoculation ring to pick out single colonies with different morphology. Finally, we isolated these single colonies by streak plate separately and repeated several times until we got the pure strains of bacteria (Fig. 2).

#### Basal identification

Gram staining^33^ is a commonly used method for classifying bacteria, and by observing the reaction of bacterial cells during the staining process, we can preliminarily determine the characteristics of their cell walls. Specific steps are preparation: smear, drying, fixation; Initial staining: drop of crystal violet staining for 1–2 min, water washing; Mordant washing: rinse off residual water with iodine solution and cover with iodine solution for one minute; Decoloration: drop of 95% alcohol, decoloration for 30 s, wash immediately; Re-staining: re-staining with sapphire red solution for 1–2 min, wash; Microscopic examination: after drying, observe with oil microscopely low magnification and then high magnification. After using an oil mirror, the lens should be wiped clean with mirror paper in time to avoid the residue of cedar oil. Additionally, physiological and biochemical properties of the strain can be identified, such as methyl red test, starch hydrolysis, gelatin liquefaction, lactose fermentation, hydrogen sulfide production, oxidase activity, and contact enzyme experiments, among others^34^.

#### Genus identification

**Figure 3:**
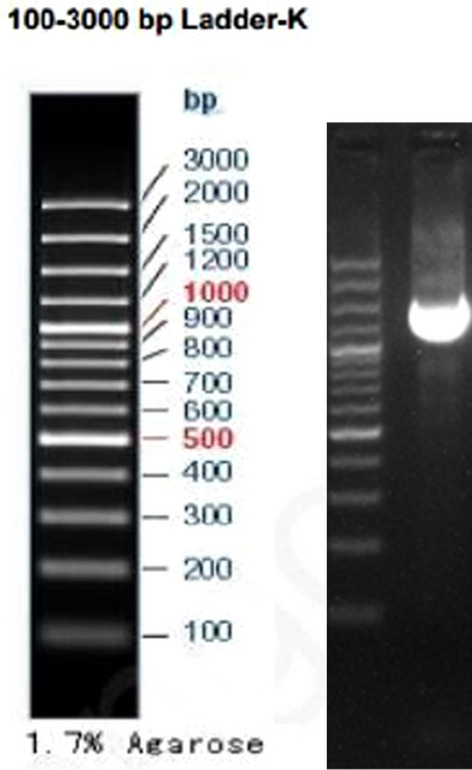
M_07_ PCR amplification electropherograms.

16S rDNA sequences^35^ are commonly used as marker gene sequences in bacterial taxonomy and phylogenetic studies. We sent the strain of bacteria to a biotechnology company, extracted DNA from M_07_, performed 16S rDNA polymerase chain reaction (PCR) amplification (Fig. 3), and sequenced the amplified product. Blast comparison of the obtained 16S rDNA sequences allowed comparison with known sequences and determination of the affinities of the strains of bacteria. A phylogenetic evolutionary tree is constructed based on the comparison results. We compared the sequences of the sequenced strain M_07_ with those of NCBI, and a phylogenetic tree was drawn using MEGA^33^ (https://www.megasoftware.net/) software for multiple sequence comparisons. The phylogenetic tree can show evolutionary relationships and genetic connections among different strains of bacteria.

### Determination of methane degradation rate of strains

#### Strains of bacteria culture and preparation of bacterial liquid

We transferred the obtained strains of bacteria into 100 mL liquid basal medium and cultured it at 36 °C and 150 r/min for 1 day to prepare bacteria solution. Then, we poured the bacteria solution into a centrifuge tube for centrifugation, added the appropriate amount of sterilized saline, and shook well. Finally, we calibrated the concentration of the bacterial solution using a spectrophotometer, obtaining an OD_600_ reading of 1.

#### Constructing the degradation system

We inoculated the prepared bacteria solution into bottles containing 50 mL of NMS medium according to the inoculum volume of 10% (v/v). For each strain of bacteria, we set up three sets of parallel experiments and, at the same time, three sets of blank control groups (without bacterial inoculation). The bottles were sealed using rubber stoppers and filled with 20% (v/v) methane.

#### Determination of degradation rate

**Figure 4:**
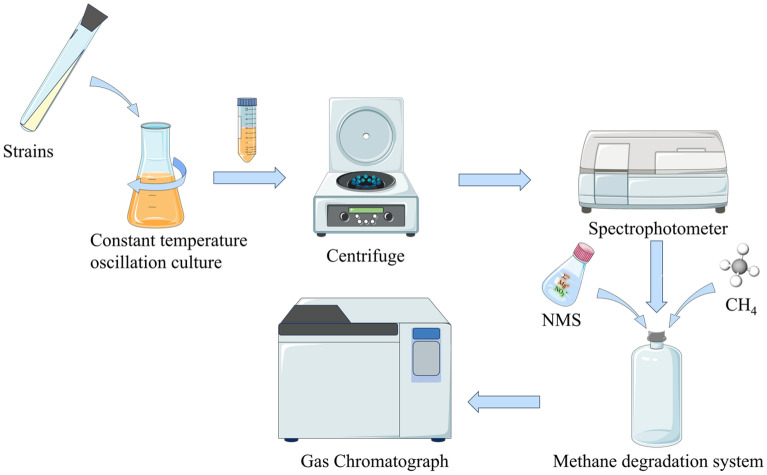
Schematic diagram of methane degradation measurement process.

Initial methane and carbon dioxide concentrations in the bottles were determined using a gas chromatograph. We placed the bottles in an oscillating culture at 36 °C and 150 r/min. After 5 day, the degraded concentrations of methane and carbon dioxide were determined by gas chromatography (Fig. 4). Finally, we calculated the methane degradation rate of the strain of bacteria based on the measurement results. The methane degradation rates of different strains were calculated using the degradation rate Eq. (1).

1 $${X}_{{CH}_{4}}=\left({v}_{0}-v\right)/{v}_{0}\times 100$$

${X}_{{CH}_{4}}$ (%/5 day) is the methane degradation rate; ${v}_{0}$ (ppm) is the initial methane content; $v$ (ppm) is the methane content after degradation.

The main reason for choosing 5 day of degradation in this experiment is that relevant studies have verified that the optimal degradation time for the determination of methane degradation rate is 5 day, so the methane degradation time was chosen as 5 day in this experiment for the determination of methane degradation rate of the strain of bacteria^36^.

### Strain methane degradation system pressure observations

We selected the strain with the highest degradation rate for pressure observation, and we followed the steps outlined in Section 2.3 for its culture and bacterial preparation.

#### Construction of the observation system

The experience set up three parallel experiments. The prepared bacterial solution was inoculated at a 10%(v/v) inoculum into a well-sealed glass bottle (with a U-tube). We filled the glass bottle with 50 mL of NMS medium and injected it with 20% (v/v) methane (Fig. 10). Before degradation, a certain amount of sterile water was added to fill the U-tube.

#### Observation of system pressure

The bottles containing culture medium were incubated in a constant temperature oscillating incubator culture at 36 °C and 150 r/min. After 5 day, observing the change in liquid level within the U-tube allows for a qualitative analysis of the gas pressure change inside the bottle before and after microbial degradation of methane.

### Effect of chelating wetting agents on the strain

#### Variation in strains of bacteria growth

The mass concentrations of the chelating wetting agent monomer were 0.005% (w/v), 0.03% (w/v), 0.05% (w/v), and 0.09% (w/v). We mixed the chelating wetting agent with NMS medium in a 1:1 volume ratio and added 10% (v/v) of the bacterial solution to the mixture as an experimental group. We also set the NMS medium without the chelating wetting agent as a control group. We used a spectrophotometer to determine the initial growth of the experimental and control groups. We determined the change in growth amount every 24 h. We compared the growth of the strains of bacteria under different conditions, and based on the experimental results, we made a preliminary speculation on how the chelating wetting agent affects the growth of the bacteria.

#### Changes in methane degradation rate

We mixed different concentrations of chelating wetting agents (0.005% (w/v), 0.03% (w/v), 0.05% (w/v), 0.09% (w/v)) with NMS medium in a 1:1 ratio. We also set up the inoculated bacteria group as a control group and the non-inoculated bacteria group as a blank control group. Six groups were set up, with three parallel experiments conducted in each group. The bottles were sealed with rubber stoppers and passed through 20% (v/v) methane. The initial concentrations of methane and carbon dioxide in the bottles were determined using gas chromatography. After incubating the bottles in a constant temperature oscillating incubator at 36 °C and 150 r/min for 5 day, we measured the concentrations of degraded methane and carbon dioxide using gas chromatography. We calculated the methane degradation rate based on the measurement results and compared the methane degradation ability of the strains after adding different concentrations of chelating wetting agents. The calculations also made it possible to quantify the changes in carbon dioxide and the relationship between methane and carbon dioxide.

## Results and discussion

### Strains enrichment, isolation, purification and identification

**Figure 5:**
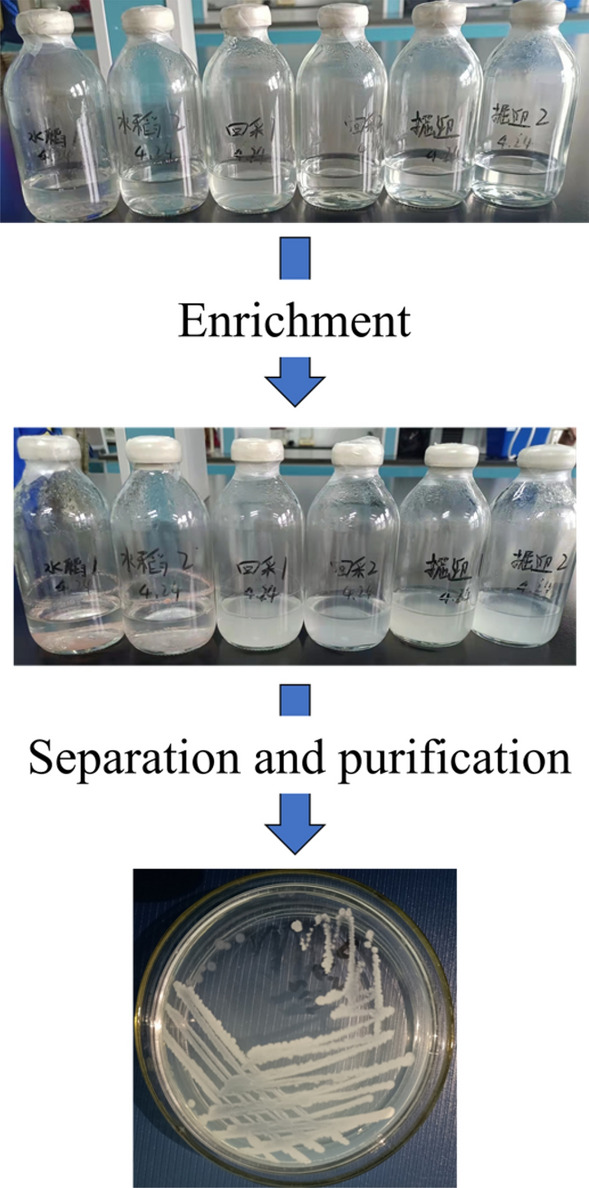
Enrichment, separation and purification results.

After 40 days of enrichment, isolation, and purification (Fig. 5), a parthenogenetic methane-oxidizing bacterial strain with the highest degradation rate (Determination of methane degradation rate of strains) that could be cultivated in basal medium was selected. This strain was named M_07_ and will be referred to by this name hereafter. The use of basal medium for its isolation and purification makes M_07_ easier to preserve compared to traditional MOB^37^. Furthermore, M_07_ exhibits a broader range of growth conditions, making it more suitable for engineering applications than conventional MOB.

**Figure 6:**
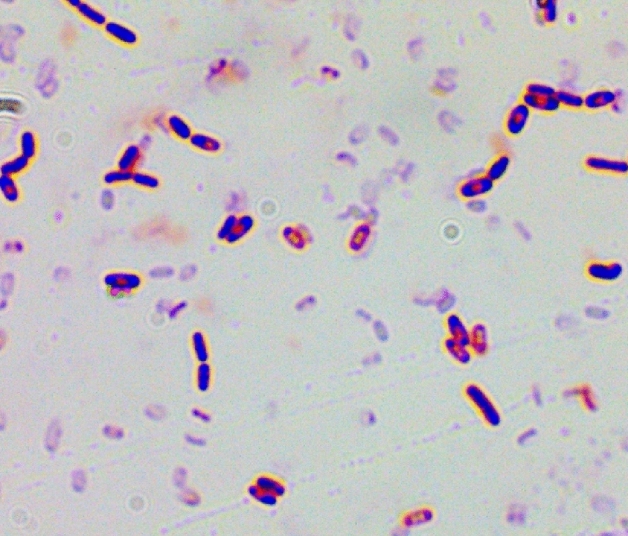
Gram staining diagram.

Based on the Gram staining result (Fig. 6), observed strain M_07_ stained purple, indicating its classification as a Gram-positive bacterium. Gram-positive bacteria, including M_07_, are known to exhibit stronger resistance to stress compared to Gram-negative bacteria. Additionally, the physiological and biochemical results (Table 1) revealed that strain M_07_ possesses the ability to produce oxidase, amylase, and pyruvate decarboxylase. Moreover, it has the capability to decompose sulfur-containing organic matter and generate hydrogen sulfide.

**Table 1:** Physiological and biochemical results.

Oxidase	Contact enzyme	Citrate	Lactose fermentation	Methyl red	Starch hydrolysis	Gelatin liquefaction	V–P	Hydrogen sulfide
+	−	−	−	−	+	−	+	+

*V–P* Voges–Proskauer.

**Figure 7:**
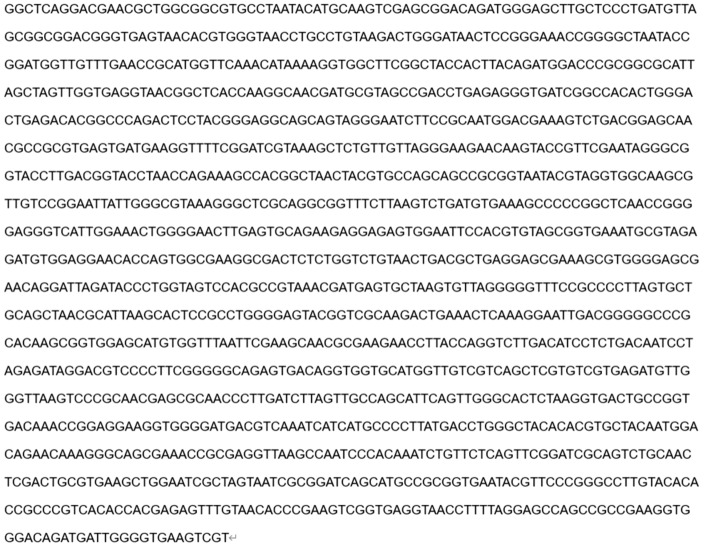
16S rDNA sequencing results.

**Figure 8:**
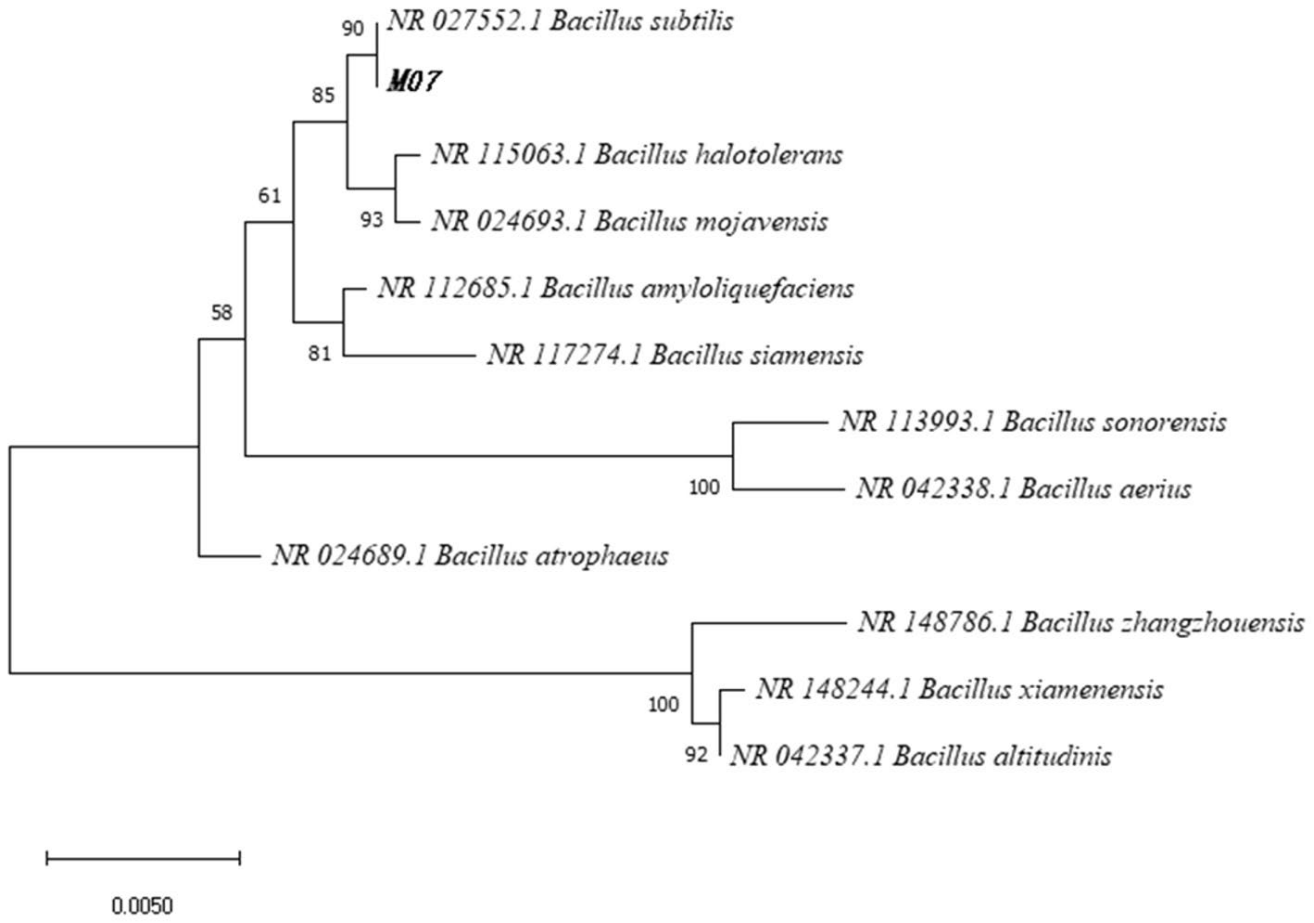
M_07_ phylogenetic evolutionary tree.

After 16S rDNA sequencing (Fig. 7) and evolutionary tree results (Fig. 8), this strain is most closely related to *Bacillus subtilis* (*NR027552.1 Bacillus subtilis*) in the *Bacillus *sp. Therefore, we identified strain M_07_ as a *Bacillus* sp*.*

As strains in the Bacillus sp. possess spore structures, it is recognized that spores are the most resistant cell form found in nature. Dormant spores exhibit high resistance to heat, dryness, radiation, acids, alkalis, organic solvents, and other bactericidal factors. Studies have demonstrated that only pressures up to 350 MPa can inactivate *Bacillus subtilis*^38^. Since strain M_07_ belongs to the *Bacillus subtilis*. Thus, compared to other MOB, strain M_07_ exhibits higher pressure resistance and tolerance, making it capable of surviving in underground environments of coal mines. However, the degree of pressure tolerance may vary depending on different experimental conditions. Nevertheless, experiments indicate that the strain can withstand higher pressure and survive under high-pressure conditions. Further experimental verification is needed to determine the actual pressure that M_07_ can withstand in coal seams water injection. In field water injection experiments, the water injection pressure is typically maintained at 20 MPa, while *Bacillus subtilis* strain M_07_ can withstand even higher pressures. It provides potential possibilities for keeping the activity of the strain in high-pressure environments during subsequent field coal seams water injection experiments.

### Determination of methane degradation rate of strains

**Figure 9:**
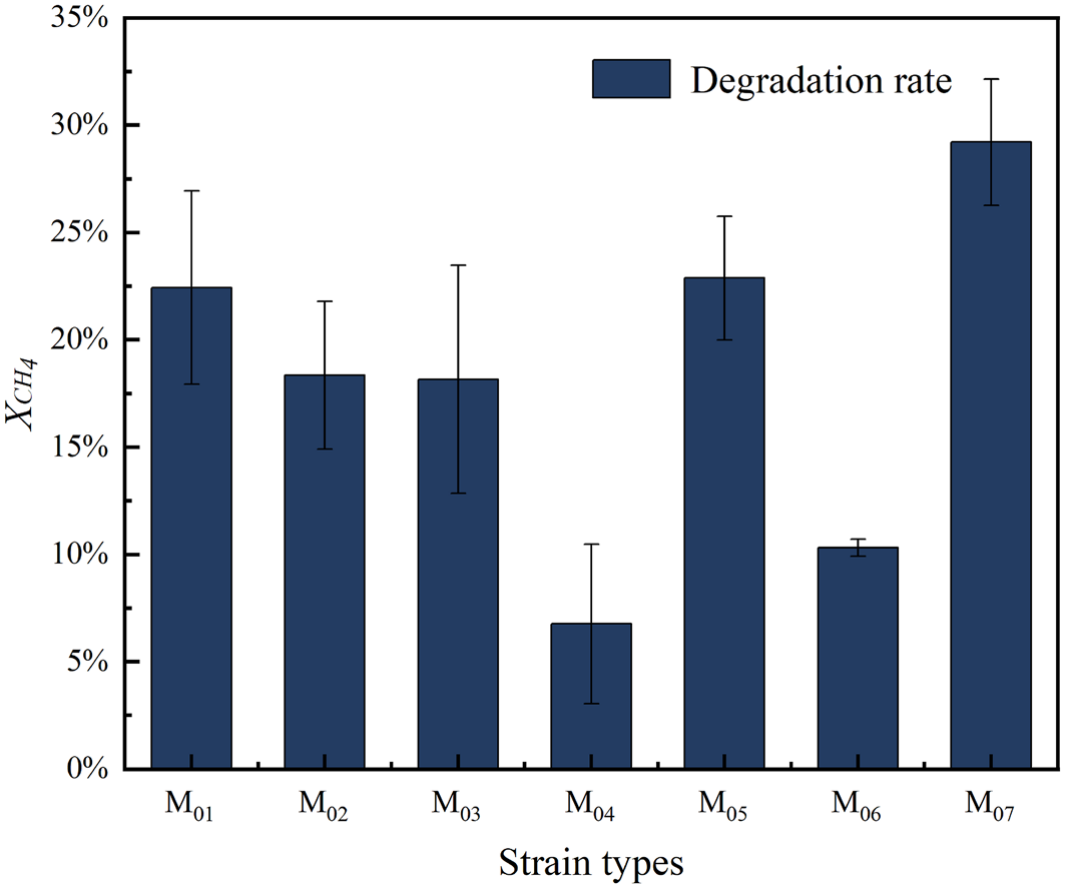
Degradation of methane by 7 strains of bacteria.

Following the enrichment, isolation, and purification of the samples, a total of seven strains, namely M_01_-M_07_, were screened out, and their methane degradation rates were determined. Based on the results (Fig. 9), it is evident that strain M_07_ exhibited the highest methane degradation rate among the seven strains. When the concentration of the calibrated bacterial solution was OD_600_ = 1, and the inoculum was 10%(v/v), strain M_07_ demonstrated a methane degradation rate of 30%. This significant finding indicates that strain M_07_ possesses a superior efficiency in degrading methane compared to the other six screened strains. Furthermore, its degradation capacity was sufficient for meeting the requirements of our laboratory. As a result, we selected strain M_07_ for further studies.

### Strain of bacteria methane degradation system pressure observations

**Figure 10:**
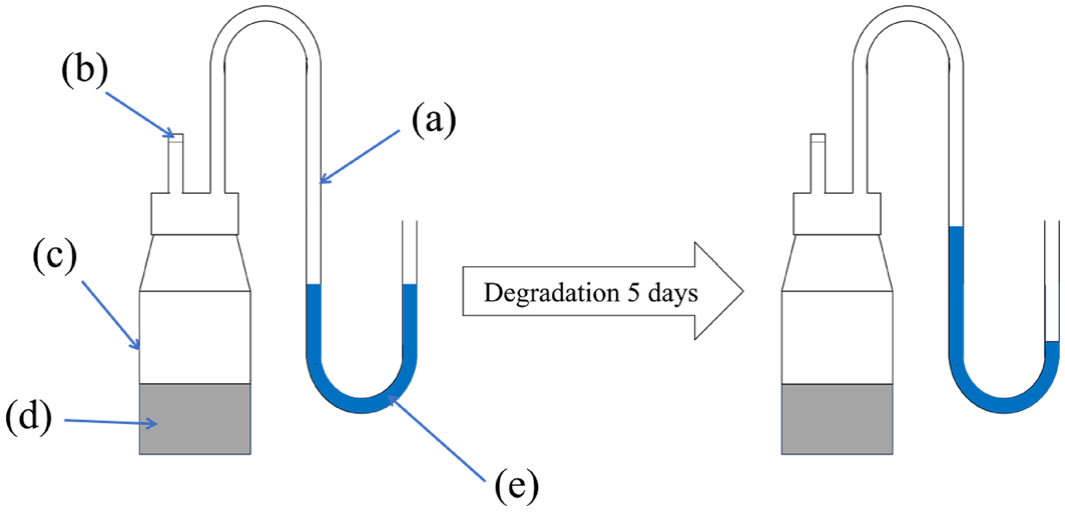
Schematic of pressure changes before and after qualitative analysis of microbial degradation of methane (**a**) U-tube; (**b**) Check valve; (**c**) Glass bottle; (**d**) NMS medium; (**e**) Distilled water.

The experimental design included a setup to examine the changes in gas pressure during methane degradation (Fig. 10). The observed movement of the liquid column towards the bottle indicated a gradual decrease in gas pressure within the bottle during the degradation of methane by M_07_. We used a U-tube with an inner diameter of 15 mm, i.e., a radius of 0.75 cm, and by measuring the height difference of the U-tube page after methane degradation, we obtained a height difference of 4.3 cm. Therefore, the cross-sectional area of the U-tube was 1.766 cm^2^, and the volume change was found to be 7.595 cm^3^. This observation further confirms that methane degradation by MOB leads to a reduction in gas pressure. During the experiment, as M_07_ degraded methane, the methane was transformed into carbon dioxide and other metabolites. Consequently, as the gas inside the bottle underwent degradation, less gas was produced, resulting in a decrease in the volume of gas within the bottle and gas pressure. Therefore, based on the results obtained and the observed phenomenon of the liquid column movement, we can qualitatively conclude that gas pressure decreases during the degradation of methane by MOB. This phenomenon further verifies the characteristic of the small amount of gas produced following methane degradation by M_07_. Additionally, it provides experimental evidence for the potential application of this strain in the field of coal and gas control and prevention.

The theoretical methane and carbon dioxide changes were obtained from the formulas.

2 $${m}_{0}=\rho \times {v}_{0}$$

3 $${c}_{0}={m}_{0}\div v$$

4 $$\updelta =\Delta c\div {c}_{0}$$

5 $$\Delta m=\Delta c\times v$$

6 $$\Delta n=\Delta m\times M$$

**Figure 11:**
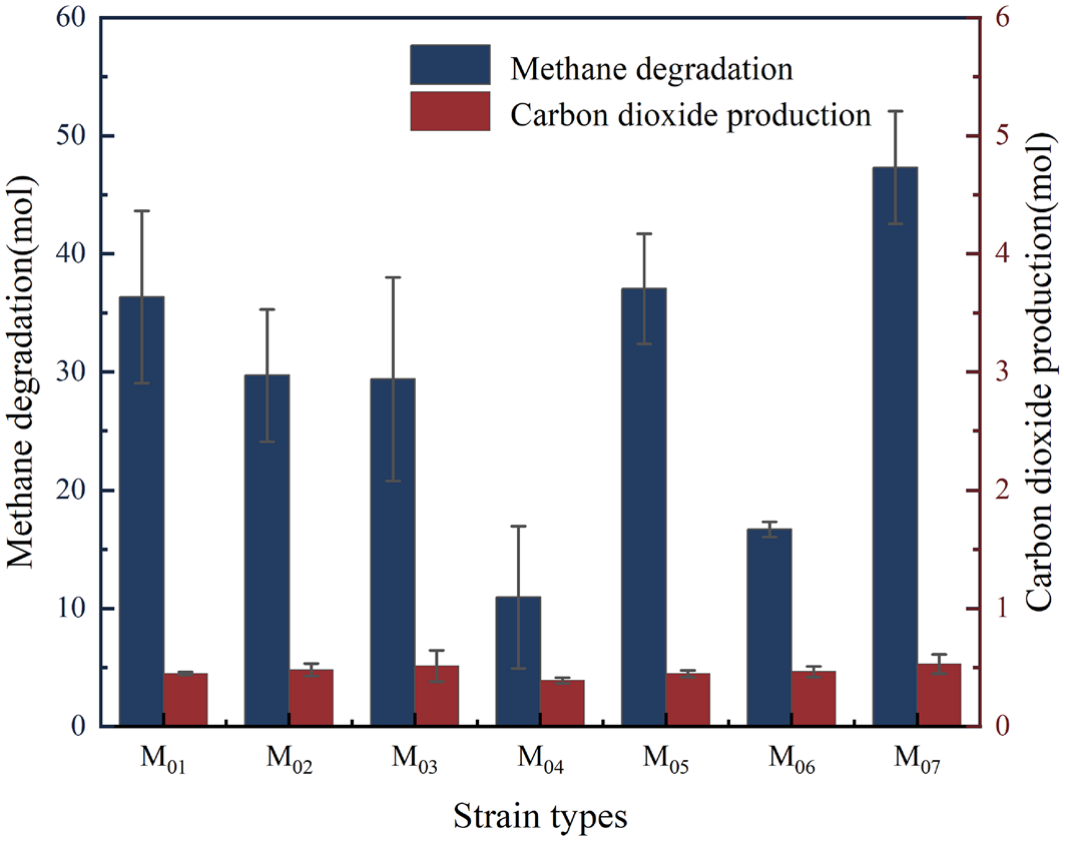
Effect of strain type on changes in methane and carbon dioxide concentrations.

${m}_{0}$: Initial mass of gas (CH_4_, CO_2_); $\rho$: Gas density; ${v}_{0}$: Initial volume of gas; ${c}_{0}$: Initial concentration of gas; $v$: Total volume of gas in the bottle; $\updelta$: Rate of change of gas concentration (%); $\Delta c$: Change in gas concentration; $\Delta n$: Amount of substance; $\Delta m$: Change in gas mass; $M$: Molar mass;

From formula (2) we get ${m}_{0}$, from formula (3) we get ${c}_{0}$, from formula (4) we get $\Delta c$, from formula (5) we get $\Delta m$, and from formula (6) we get $\Delta n$. At last, we get the effect of strain type on changes in methane and carbon dioxide concentrations (Fig. 11).

**Figure 12:**
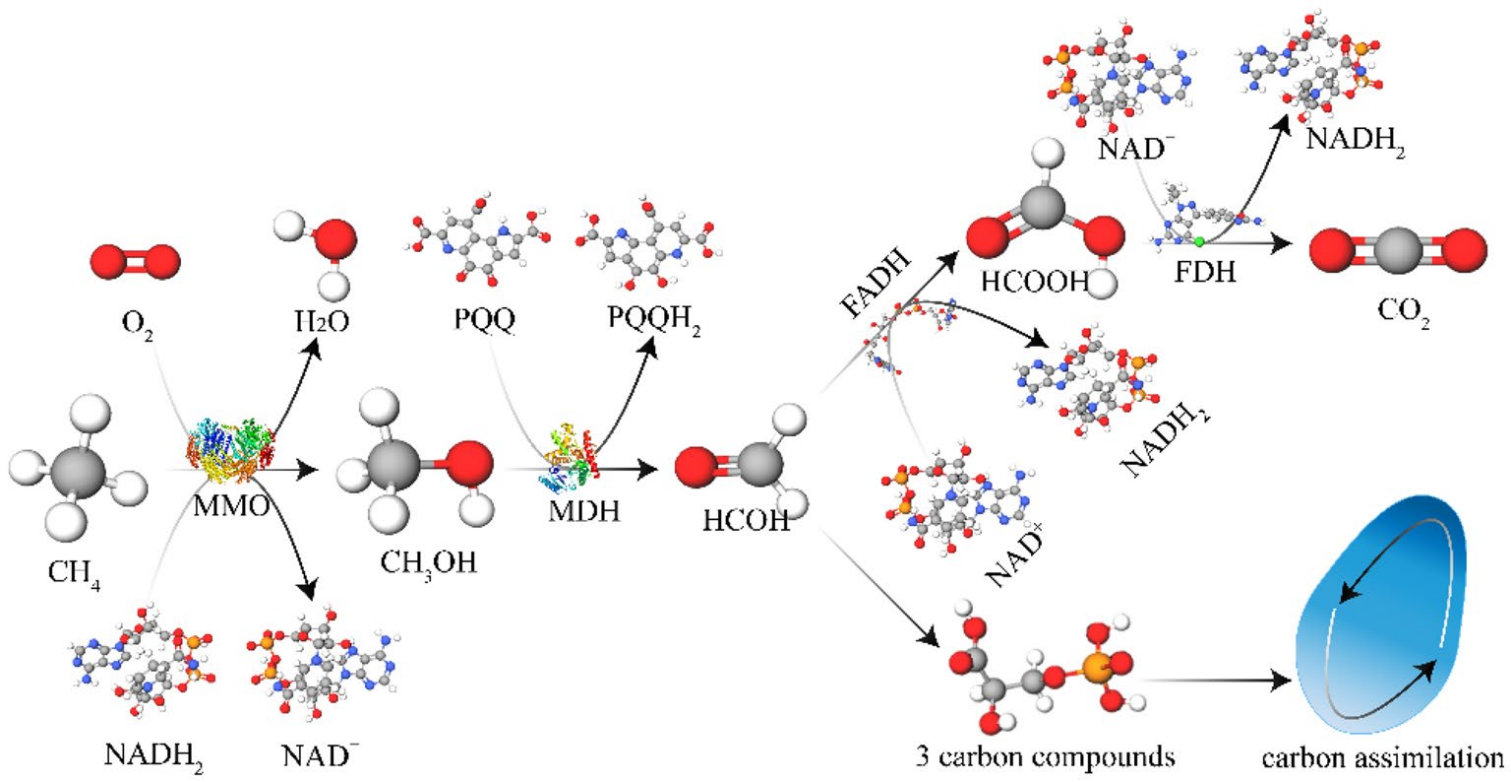
Diagram of the mechanism of carbon dioxide production from methane degradation by MOB. *MMO* methane monooxygenase, *MDH* methanol dehydrogenase, *FADH* formaldehyde dehydrogenase, *FDH* formaldehyde dehydrogenase, *PPQ* pyrroloquinoline quinine, *PPQH*_*2*_ quinol form, *NAD* oxidized forms nicotinamide adenine dinucleotide, *NADH*_*2*_ reduced forms of the nicotinamide adenine dinucleotides.

Based on the comparison results (Fig. 11), it is evident that all seven strains degraded a significant amount of methane while producing only small amount of carbon dioxide during the degradation process. We can infer the relationship between methane degradation and carbon dioxide production from the concentration of these gases in the system. Strain M_07_ degraded 70 mol of methane to produce 1 mol of carbon dioxide. This phenomenon can be attributed to the presence of methane monooxygenase (MMO) in the *Bacillus* sp.^39^. MMO oxidizes methane to methanol, which is further oxidized to formaldehyde by methanol dehydrogenase (MDH)^40^. A portion of the formaldehyde produced is assimilated into cellular material, while the rest is oxidized to carbon dioxide and released from the cell^41^. This process indicates that only small amount of gas is produced after methane degradation by MOB, with most being converted to biomass. Thus, it leads to a reduction in gas pressure (Fig. 12). These results confirm the phenomenon of reduced gas pressure in the system before and after methane degradation.

By applying M_07_ to water injection in coal seams gas pressure in the coal seams reduces, thereby preventing gas protrusion. However, further experimental studies are needed to investigate the degradation mechanism of methane by M_07_ and the quantitative relationship between methane and carbon dioxide.

### Effect of chelated wetting agents on the growth of M_07_

**Figure 13:**
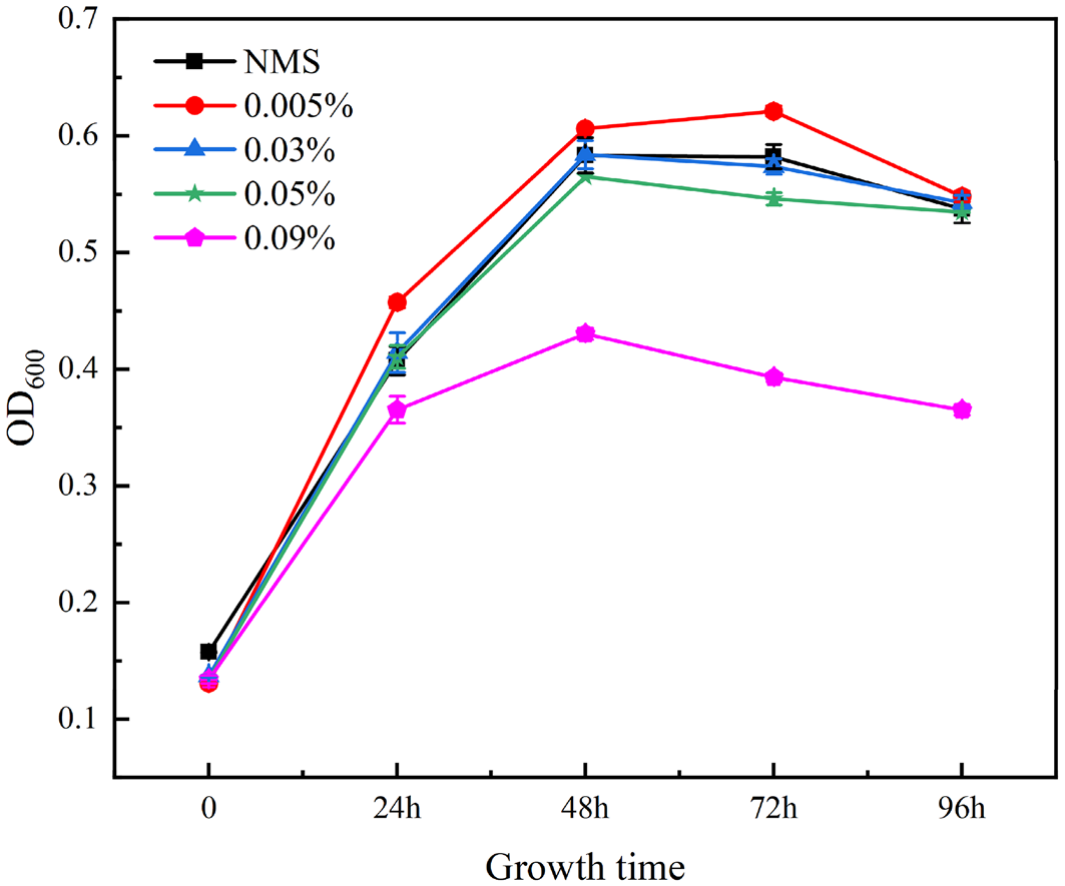
Changes in the growth of M_07_ at different concentrations. NMS: y = − 0.000095 x^2^ + 0.013003 x + 0.157335, R^2^ = 0.993187; 0.005%: y = − 0.000116 x^2^ + 0.015267 x + 0.139650, R^2^ = 0.995021; 0.03%: y = − 0.000099 x^2^ + 0.013521 x + 0.142418, R^2^ = 0.989287; 0.05%: y = − 0.000093 x^2^ + 0.012821 x + 0.143599, R^2^ = 0.981196; 0.09%: y = − 0.000077 x^2^ + 0.009433 x + 0.150885, R^2^ = 0.942935.

The growth of strain M_07_ was monitored every 24 h across different experimental groups. By comparing the growth of M_07_ under varying concentrations of a chelating wetting agent (Fig. 13), it is evident that the growth trends follow a similar pattern over time, characterized by an initial increase followed by a subsequent decrease. This phenomenon can be analyzed as follows: in the early stages, the culture medium provided sufficient nutrients, including the required nitrogen and carbon sources for MOB in the bottle. At this stage, the microbial population was small, and there was ample space for their growth. So, the growth of MOB from 0 to 48 h showed an increasing trend. However, as the growth of MOB consumed the nutrients in the bottle, the carbon and nitrogen sources on the bottle were progressively reduced, becoming insufficient to support massive bacteria propagation. Consequently, the number of MOB stabilized. The continuous consumption of nutrients was not enough to maintain the continued growth of MOB, leading to their death due to the decrease of nutrients and the increase of their own by-products, such as carbon dioxide. Hence, the growth of MOB decreased gradually from 48 to 96 h. Therefore, irrespective of whether the chelating wetting agent was added or not and regardless of the concentration added, the growth of MOB exhibited a trend of increasing first and then decreasing.

The extreme values of MOB grown at different chelating wetting agent concentrations and their corresponding times were calculated by polynomials (Table 2). Analyzing the reasons for the change in extreme value and time, IDS promotes microbial growth when the concentration is 0.005%, so the extreme value becomes higher, and the time point corresponding to the extreme value of growth is shifted forward. After that, with the increase of concentration, the growth extreme gradually becomes smaller and the time increases, but to 0.09% concentration, the growth extreme is the smallest, and the time point corresponding to the extreme is shifted forward, analyzing the main reason may be due to the toxicity is the largest at this concentration, which leads to the destruction of the cellular structure and even the death in the late stage, so its growth decreases and the time of the extreme point appears in advance.

**Table 2:** Extreme values of different concentrations of chelating wetting agents and their corresponding time.

Chelating wetting agent concentration	Time (h)	OD_600_
NMS	68.4368	0.6023
0.005%	65.8060	0.6420
0.03%	68.2879	0.60413
0.05%	68.9301	0.5855
0.09%	61.2532	0.4398

### Effect of chelating wetting agents on the degradation rate of M_07_ methane

**Figure 14:**
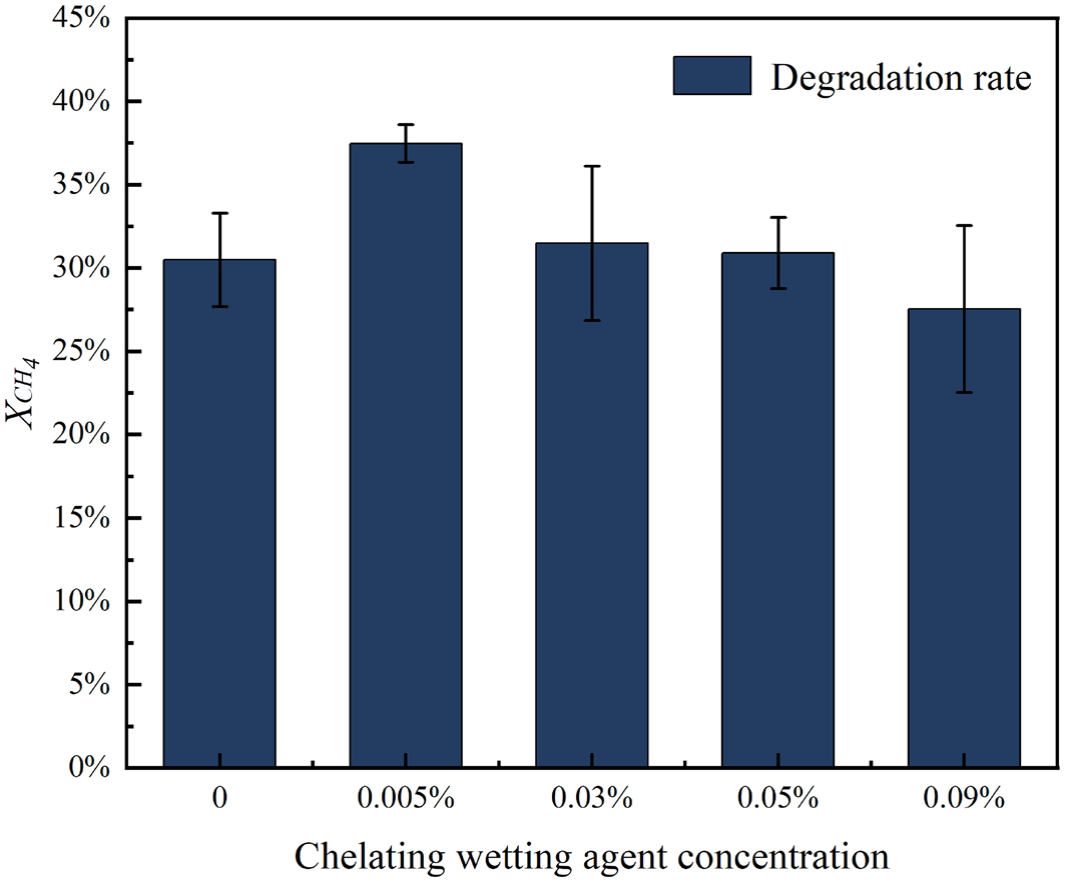
Degradation of methane by M_07_ at different concentrations.

We prepared a mixture of chelating wetting agents and strain M_07_, measuring the degradation rate of M_07_ at various concentrations. Then, we compared the degradation rate of the M_07_ when grown solely in NMS medium (Fig. 14).

Based on the experimental findings, it was observed that the methane degradation rate of strain M_07_ exhibited an increasing and then decreasing trend with the concentration of the chelating-type wetting agent. Specifically, at a concentration of 0.005% (w/v), the degradation rate reached 37.47%, showing a 22.93% increase compared to the degradation rate without the chelating-type wetting agent. At a concentration of 0.03% (w/v), the degradation rate increased by 3.31%, and at 0.05% (w/v) concentration, the degradation rate remained relatively unchanged. However, at a concentration of 0.09% (w/v), the degradation rate decreased by 9.68%. This pattern of change aligns with the growth trend of strain M_07_, indicating a correlation between its growth and methane degradation capacity. When the growth amount of M_07_ was higher, its methane degradation ability also increased, whereas the lower growth amount resulted in a corresponding decrease in methane degradation ability. The chelating wetting agents influenced the growth of MOB, thereby affecting their methane degradation rate. It may be due to the effect of IDS and SDBS on the growth of MOB, which leads to changes in their enzyme activity, hence their degradation rate. An appropriate amount of surfactant promotes the growth and metabolic activity of microorganisms, enhancing the degradation ability of MOB. However, excessive surfactant amounts have detrimental effects on microorganism growth and metabolic activity, damaging cellular barriers and hindering growth, ultimately leading to reduced methane degradation ability^42^. Thus, based on the experimental results, when the concentration of the chelating wetting agent is below 0.05% (w/v), it promotes the enzyme activity of MOB, thereby enhancing their metabolic activity and improving methane degradation ability. Conversely, when the concentration exceeds 0.05%(w/v), it inhibits the enzyme activity of MOB, reducing their metabolic activity and consequently decreasing their methane degradation ability.

Based on the experimental results examining the growth and methane degradation of the strain, a chelating wetting agent concentration of 0.005% (w/v) was found to be the most effective in enhancing both. The results of the current experiments have demonstrated that a chelating wetting agent concentration of 0.005% (w/v) is the most suitable concentration for strain M_07_. The degradation rate of M07 at 0.005% concentration was 22.93% higher than that without the addition of the chelating wetting agent. Therefore, it is feasible to study methane degradation by MOB based on chelating wetting agent carriers. This bacterial agent can be used as a new technology to prevent and control coal and gas outbursts in deep coal seams, and it will have a better application prospect in the deep low-permeability coal seams in the future.

## Conclusions and outlook

In this paper, seven strains of MOB were screened based on the coal mine. The strain M_07_ with the optimal degradation rate as the subsequent experimental object, which was further mixed with the chelating wetting agent to determine the growth and degradation rate changes of strain M_07_ at different concentrations, and the conclusions are as follows:

The strain M_07_, screened from coal mines, is more suitable for survival in coal mines. M_07_ belongs to the genus *Bacillus*, which is a Gram-positive bacterium with stronger resistance and adapted to high-pressure water injection conditions.

When M_07_ degraded 70 mol of methane, it produced 1 mol of carbon dioxide, and most of the methane was eventually converted to cytoplasm, which could reduce the gas pressure in the system.

Concentrations of chelating wetting agents below 0.05% (w/v) promote the growth of M_07_ and increase the methane degradation rate. The highest degradation rate of 37.47% was achieved at a concentration of 0.005% (w/v). However, exceeding 0.05% (w/v) inhibits its growth and reduces the methane degradation rate. Therefore, the chelating wetting agent concentration of 0.005% (w/v) is the more suitable concentration for strain M_07_ to maximize its methane degradation.

The microbial agent of the chelating wetting agent carrier can play the roles of MOB and chelating wetting agent at the same time, improving the wettability of the coal seams, and the pore connectivity better, to promote the MOB to inject into the coal seam better, reduces the gas pressure and content of the coal seams. At the same time, it can prevent and control the occurrence of coal and gas outbursts, and provide a new method for the prevention and treatment of the disaster in the deep low-permeability coal seams.

However, since this study is still in its early stages, further research is required in the following areas: (1) Scholars have examined the degradation mechanism of *Bacillus subtilis*, but further investigation is required to determine its applicability in extreme coal mine environments. (2) While MOB can reduce environmental gas pressure and aid in gas management, it is necessary to study the actual changes in gas pressure during water injection in coal mines. (3) MOB can prevent the spontaneous combustion of coal seams by using oxygen in coal seams theoretically, and the solution to the spontaneous combustion of coal seams by MOB needs to be researched. (4) The carrier of microbial preparation is a chelating wetting agent, and it is necessary to explore the type, concentration, and proportion of a better carrier. To comprehensively prevent and control the compound dynamic disaster in deep low-permeability coal seams.

## Data Availability

The datasets generated and/or analysed during the current study for strain M_07_ are available in the NCBI GenBank repository, accession number OR883907.1, https://www.ncbi.nlm.nih.gov/nuccore/OR883907.1. All the remaining data supporting the findings of this study are available within the manuscript.
